# Anticancer Effect of* Nemopilema nomurai* Jellyfish Venom on HepG2 Cells and a Tumor Xenograft Animal Model

**DOI:** 10.1155/2017/2752716

**Published:** 2017-07-13

**Authors:** Hyunkyoung Lee, Seong Kyeong Bae, Munki Kim, Min Jung Pyo, Minkyung Kim, Sujeoung Yang, Chung-kil Won, Won Duk Yoon, Chang Hoon Han, Changkeun Kang, Euikyung Kim

**Affiliations:** ^1^Institute of Animal Medicine, Gyeongsang National University, Jinju 660-701, Republic of Korea; ^2^College of Veterinary Medicine, Gyeongsang National University, Jinju 660-701, Republic of Korea; ^3^Department of Anatomy, College of Veterinary Medicine, Gyeongsang National University, Jinju 660-701, Republic of Korea; ^4^Headquarters for Marine Environment, National Fisheries Research & Development Institute, Shiran-ri, Gijang-eup, Gijang-gun, Busan 619-705, Republic of Korea

## Abstract

Various kinds of animal venoms and their components have been widely studied for potential therapeutic applications. This study evaluated whether* Nemopilema nomurai *jellyfish venom (NnV) has anticancer activity. NnV strongly induced cytotoxicity of HepG2 cells through apoptotic cell death, as demonstrated by alterations of chromatic morphology, activation of procaspase-3, and an increase in the Bax/Bcl-2 ratio. Furthermore, NnV inhibited the phosphorylation of PI3K, PDK1, Akt, mTOR, p70S6K, and 4EBP1, whereas it enhanced the expression of p-PTEN. Interestingly, NnV also inactivated the negative feedback loops associated with Akt activation, as demonstrated by downregulation of Akt at Ser473 and mTOR at Ser2481. The anticancer effect of NnV was significant in a HepG2 xenograft mouse model, with no obvious toxicity. HepG2 cell death by NnV was inhibited by tetracycline, metalloprotease inhibitor, suggesting that metalloprotease component in NnV is closely related to the anticancer effects. This study demonstrates, for the first time, that NnV exerts highly selective cytotoxicity in HepG2 cells via dual inhibition of the Akt and mTOR signaling pathways, but not in normal cells.

## 1. Introduction

Hepatocellular carcinoma (HCC) is one of the most deadly cancers owing to its high rates of recurrence and metastasis [1]. HCC-related mortality is especially high in Asian and African countries. According to results published by GLOBOCAN 2012, the incidence of HCC in Korea is the sixth highest worldwide [2]. Despite advances in diagnostic imaging techniques and management of HCC, most patients with HCC are diagnosed with advanced-stage disease, which is regarded as incurable [3]. Chemotherapy for HCC is not satisfactory and the survival rate is extremely poor. Until now, the US Food and Drug Administration (FDA) has only approved one systemic treatment, sorafenib. Therefore, novel and more effective therapies for advanced HCC are necessary.

Venoms have attracted the attention of researchers engaged in identifying active components and developing novel drug candidates owing to their high sensitivity and specificity for target molecules. They have been used in traditional medicine, mainly in Asia and Africa. Cobra venom has been used to treat joint pain, inflammation, and arthritis in Indian traditional medicine, “Ayurveda” [4]. Bee venom has also been used to treat chronic inflammation (rheumatoid arthritis) and skin disease (acne and itch) and as pain relief for thousands of years [5, 6]. Recently, drugs derived from venom have been developed and are being used for treating patients suffering from various diseases [7–10]. To date, there are six FDA-approved drugs derived from venoms and another ten drugs are being tested in clinical trials.

Recently, jellyfish populations have dramatically increased owing to global warming and changes in the salinity of oceans. Jellyfish have nematocysts, which are specialized venom-containing cells in the tentacles. Under some stimuli or conditions involving skin contact, a nematocyst is rapidly discharged, which penetrates victims and delivers venom. Jellyfish venom is composed of various chemicals, including peptides, enzymes, neurotoxin, cytolysin, and hemolysin [11–17]. Jellyfish are regarded to be hazardous to other forms of life and cause waste in marine environments as they damage the fishery industry and cause health problems in humans [18, 19]. However, recently, the potential bioactivity of jellyfish has been reported to exhibit insecticidal, immunostimulative, anticoagulant, and antihypertensive effects [20–23]. Active components isolated from* Rhopilema esculentum* jellyfish venom have been demonstrated to have insecticidal activities [23].* Aurelia aurita* venom shows anticoagulant effects through strong fibrinogenolytic activity by cleaving the A*α* and B*β* chains of fibrinogen molecules [24]. Therefore, jellyfish venom is an interesting and potentially useful resource.* Nemopilema nomurai,* one of the largest jellyfish species, can grow up to a diameter of 2 m and a weight of 200 kg. They are widely distributed and bloom off the coasts of Korea, China, and Japan [19]. It has been reported that* N. nomurai* has antioxidant activity, so it is a potentially beneficial food for humans [20]. A collagen extract from* N. nomurai* can stimulate the production of immunoglobulin and cytokines without causing any allergic complications, indicating that it has a regulatory effect on the immune system [21]. Qniumucin, a glycoprotein derived from* N. nomurai* jellyfish, was found to inhibit degeneration of articular cartilage in an in vivo osteoarthritis model [22]. However, there is little information about the anticancer effect of* Nemopilema nomurai* venom. The present study explored the novel pharmacological effects of NnV on cancer cells, its anticancer activity, and its underlying molecular mechanisms of action.

## 2. Materials and Methods 

### 2.1. Chemicals and Reagents

Bovine serum albumin (BSA), Dulbecco's Modified Eagle's Medium (DMEM), fetal bovine serum (FBS), penicillin, RPMI-1640, streptomycin, and trypsin were purchased from Gibco-BRL (Grand Island, NY, USA). Dimethyl sulfoxide (DMSO), 3-(4,5-dimethylthiazol-2-yl)-2,5 diphenyltetrazolium bromide (MTT), and propidium iodide (PI) were from Sigma-Aldrich Inc. (St. Louis, MO, USA). Antibodies for caspase-7, caspase-9, caspase-3, Bcl-xL Mcl-1, PARP, Bcl-2, p-Akt, p-PI3K, p-PDK, p-PTEN, p-mTOR, p-p70S6K, p-S6K, p-4EBP1, p-eIF4E, and GAPDH were obtained from Cell Signaling Technology (Beverly, MA, USA). Terminal deoxynucleotidyl transferase dUTP nick end labeling (TUNEL) System Kit was procured from Calbiochem (Darmstadt, Germany). ALT, AST, and CK were obtained from IDEXX Laboratories Inc. (Westbrook, Maine USA). All other reagents used were of the purest grade available.

### 2.2. Jellyfish Collection and Preparation

The specimens of* N. nomurai* jellyfish were captured from the Korea Strait along the coasts of Geoje in September, 2012. Only tentacles were collected and transferred immediately to a laboratory for further preparation. Nematocysts were isolated from the dissected tentacles as described in a Bloom method with slight modification [25]. In brief, dissected tentacles were rinsed with cold seawater to remove debris. The tentacles were placed in three volumes of cold seawater for 24 h with gentle swirling for 30 min once daily at 4°C. After autolysis, the supernatant was collected and centrifuged at 4,000*g* for 10 min and the settled material was resuspended in fresh seawater and set for autolysis for 24 h. This process was repeated for 3 days. The sediments were collected and centrifuged at 4,000*g* for 10 min and washed several times with fresh sea water by centrifugation at 100*g* at 4°C for 5 min until debris around nematocysts was almost removed. Finally, the undischarged nematocysts were collected, lyophilized, and stored at −70°C until use.

### 2.3. Venom Extraction and Preparation

Venom was extracted from freeze-dried nematocysts using the technique described by Seymour et al. with a minor modification [26]. In brief, venom was extracted from 50 mg of lyophilized nematocyst powder in 1 ml of cold phosphate buffered saline (PBS, pH 7.4, 4°C) using glass beads (0.5 mm in diameter). This mixture was shaken at 3000 rpm for 30 s, which was repeated for ten times with intermittent cooling on ice. The venom extracts were transferred to a new Eppendorf tube and centrifuged (15,000*g*) at 4°C for 30 min. The supernatant was used as NnV for the present study. Protein concentration of NnV was determined by Bradford (1976) method (Bio-Rad, CA, USA) [27].

### 2.4. Cell Culture

For finding the target cancer cells of NnV, various cancer cell lines were used, including A549 (human pulmonary adenocarcinoma), HepG2 (human hepatocellular carcinoma), HT-29 (human colon adenocarcinoma), MCF7 (human breast carcinoma), MDA-MB-231 (human breast adenocarcinoma), HaCaT (keratinocyte), human dermal fibroblast (HDF), H9C2 (cardiomyocyte), and WB-F344 (liver epithelial cell) cells. The A549 cell was maintained in RPMI-1640 and HDF cell was cultured in FGM™-2 BulletKit™ (Lonza Group Ltd., Basel, Switzerland) and the others were maintained in DMEM supplemented with 10% FBS and 100 *μ*g/ml penicillin-streptomycin-amphotericin B solution. They were grown as monolayer cultures and kept at 37°C in a humidified atmosphere with 5% CO_2_ for growth. WB-F344 and HaCaT cells were kindly supplied by Professor Changkeun Kang (Gyeongsang National University, Jinju, Korea). HDF cells were purchased from MCTT (Modern cell & Tissue Technologies, Inc., Seoul, Korea). Other cells used in the present study were purchased from American Type Culture Collection (ATCC).

### 2.5. Cell Viability

Cells were seeded in 24-well plates at a density of 4 × 10^4^ cells/well. After 24 h, the cells were treated for another 24 h with increasing concentrations of NnV (0–10 *μ*g/ml). MTT solution (5 mg/ml) was then added to each well and incubated for additional 3 h at 37°C. After the supernatant was removed, dimethyl sulfoxide (DMSO) was added to each well to dissolve the formazan crystal generated. Cell viability was determined by measuring optical density at 540 nm using ELISA multiwell plate reader (PowerWaveXS, BioTek Instruments, Inc., Winooski, USA).

### 2.6. Cell Cycle Analysis

Cells were seeded in 6-well plate at a density of 1 × 10^5^ cells/well a day before experiment. After the treatment of NnV for 24 h, both adherent and suspended cells were collected and pooled together for the analysis of cell cycle. The cell pellets were resuspended in 300 *μ*L of PBS and fixed by adding 700 *μ*L EtOH. The cells were incubated at −20°C overnight and washed with fresh PBS again. The cellular DNA was stained with PI (50 *μ*g/ml) in the presence of RNase A (10 *μ*g/ml). A FACSCalibur flow cytometer (BD Biosciences, Franklin Lakes, NJ, USA) was used to analyze the cell cycle distribution.

### 2.7. Nuclear Morphology

For nuclear morphological analysis, HepG2 cells (1 × 10^4^ cells/well) were plated onto cell culture slide (SPL life science, Pocheon, Korea) and treated with indicated concentrations of NnV (0–1.2 *μ*g/ml) for 24 h. The cells on slide were fixed with 4% paraformaldehyde for 15 min and washed three times for 10 min each. Then they were stained with Hoechst 33342 fluorescent dye (0.5 *μ*g/ml) for 15 min. Nuclear morphology was observed by fluorescence microscopy (Leica, Wetzlar, Germany) using a 200x objective.

### 2.8. Western Blot Analysis

Cells were seeded in 6-well plate and were incubated with various concentrations of NnV for 24 h. The treated cells were collected by scraping with 150 *μ*L of RIPA buffer containing protease inhibitor cocktail. Protein sample concentration was measured using the Bradford protein assay (Bio-Rad). Lysates (50 *μ*g) were separated by 12% SDS-polyacrylamide gel and then transferred into PVDF membranes (Bio-Rad, CA, USA). Western blots were probed with specific primary antibodies overnight at 4°C. Following incubation with horseradish peroxidase-conjugated secondary antibody (Cell Signaling Technology, Beverly, MA) for 1 h at room temperature, the blots were visualized by using an enhanced chemiluminescence method (ECL, Amersham Biosciences, Buckinghamshire, UK) and analyzed using ChemiDoc XRS (Bio-Rad, CA, USA). Densitometry analysis was performed with a Hewlett-Packard scanner and NIH Image software (ImageJ).

### 2.9. Animals and Tumor Xenograft Model

Male athymic nude mice at the age of 4 weeks were purchased from the Central Lab Animal Incorporation (Seoul, Korea) and housed in the Animal Resource Facility at the Gyeongsang National University at Jinju in accordance with good animal practice as defined by the NIH guidelines. The animal protocol used in this study was approved by the Institutional Animal Care and Use Committee of the Gyeongsang National University at Jinju, and the animal protocol number is GNU-130129-M0018.

HepG2 cells (1 × 10^7^ in 200 *μ*L PBS) were injected subcutaneously into the right flank of each mouse. When the tumor volume reached over 100 mm^3^, the animals were divided randomly into three groups with eight mice per group. The mice in control group were intraperitoneally (IP) injected with 200 *μ*L PBS once every other days for 20 days, while NnV-treated groups were injected with either NnV 0.1 or 0.3 mg/kg. The tumor growth and body weight of each mouse were recorded once every other days during experimental period. Tumor sizes were measured using Vernier calipers and their volumes were calculated using the hemiellipsoidal model formula: tumor volumes = [tumor length in mm × (tumor width^2^ in mm)]/2. The experiment was terminated 22 days after NnV treatment. At this time, mice were sacrificed, tumor from each mouse was excised, and wet weight of each tumor in each group was recorded. Each tumor tissue was fixed in 10% neutral buffered formalin and paraffin-embedded and used for H&E staining, TUNEL assay, and immunohistochemistry assay.

### 2.10. Biochemistry Analysis in Blood

Blood samples were collected from the caudal vena cava of the mice and then there was centrifugation at 3000 ×g for 5 min. These samples were used for determination of alanine transaminase (ALT), aspartate aminotransferase (AST), and creatinine kinase (CK) using clinical chemistry analyzer system (VetTest 8008; IDEXX Laboratories, Westbrook, ME, USA).

### 2.11. Histological Examination

Excised liver and heart from the control and NnV-treated groups were fixed in 10% neutral buffered formalin and processed into paraffin blocks. Sections were deparaffinized in xylene and rehydrated in graded-alcohol washes. H&E staining was performed using standard procedure. The specific staining was visualized and images were acquired using a light microscope with 200x objectives (Olympus, Center Valley, PA, USA).

### 2.12. DNA Fragmentation Detection in Tumor Tissue

Apoptosis was detected using a terminal deoxynucleotidyl transferase dUTP nick end labeling (TUNEL) System Kit (Calbiochem). 5 *μ*m thick tumor tissue sections were deparaffinized and rehydrated and treated with 20 *μ*g/ml proteinase K for 20 min. The DNA was end-labeled with Biotinylated Nucleotide Mix in terminal deoxynucleotidyl transferase buffer at 37°C for 30 min and visualized by inverted fluorescence microscopy (Leica, Wetzlar, Germany). Total cell nuclei were stained by DAPI counterstaining. The quantification of TUNEL-positive cells was performed with a Hewlett-Packard scanner and NIH Image software (Image J).

### 2.13. Immunohistochemical Analysis in Tumor Xenografts

Immunohistochemistry (IHC) for Ki-67, p-Akt, and E-cadherin was performed using rabbit monoclonal antibodies. Tumor tissues were deparaffinized with xylene twice for 5 min and rehydrated and rinsed with tap water for 10 min. They were incubated at 4°C with primary antibodies overnight. Immunoreactivity was visualized with an avidin-biotin peroxidase reaction (PK-4001, Vectastain ABC kit). The peroxidase reaction was developed using a 3,3′-diaminobenzidine tetrahydrochloride (D-5905, Sigma). The sections were counterstained with hematoxylin before being mounted.

### 2.14. Isolation of Bioactive Component with Anticancer Effect from NnV

NnV was extracted in 10 Mm Tris-HCl buffer (pH 7.8) and fractionated on DEAE cation-exchange column. The proteins were eluted with a linear gradient of NaCl (0–80%) using three column volumes of eluting phosphate buffer. The flow rate was adjusted to 1 ml/min and 1.5 ml fraction was collected in each tube. Each protein peak was checked for cytotoxicity on HepG2 cells. The fractions showing cytotoxic activity were pooled and the protein amounts were measured.

### 2.15. Statistical Analysis

The results are expressed as mean ± standard deviation (SD). One-way analysis of variance (ANOVA) was used to evaluate the significance of difference between two mean values. *p* < 0.05 was considered to be statistically significant.

## 3. Results

### 3.1. NnV Is Selectively Cytotoxic toward HepG2 Cells

**Figure 1:**
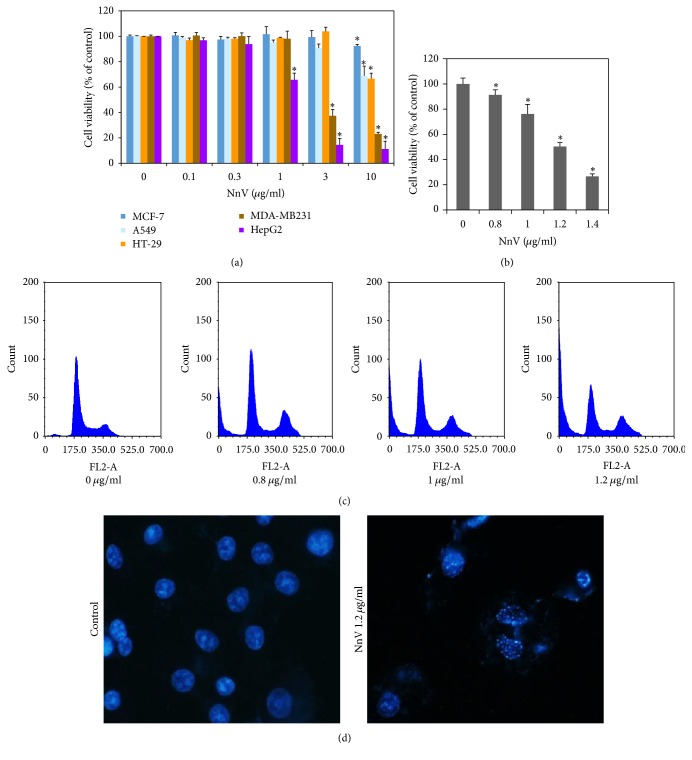
Effects of NnV on the cell viability in different cancer cells. (a) Cancer Cells (A549, HT-29, MCF-7, MDA-MB231, and HepG2 cells) were incubated for 24 h at various concentrations (0, 0.3, 1, 3, and 10 *μ*g/ml) of NnV. Cell viability was analyzed by the MTT assay. (b) To determine efficient concentrations of cytotoxicity in HepG2, the cells were incubated for 24 h with various concentrations (0–1.4 *μ*g/ml). (c) Apoptosis was measured by PI staining followed by flow cytometry analysis. (d) Hoechst 33342 fluorescence dye staining was performed and nuclear morphology was examined by fluorescence microscopy (Leica) using a 400x objective. The data shown are the mean ± SD of six independent experiments. ^*∗*^*p* < 0.05 was considered to indicate statistical significance compared with nontreated controls.

To determine the potential anticancer effect of NnV on various cancer cells, A549, HepG2, HT-29, MCF-7, and MDA-MB231 cells were treated with NnV for 24 h, and then an MTT assay was performed. The proliferation of HepG2 and MDA-MB231 was markedly suppressed, with an IC_50_ of 1.27 *μ*g/ml and 2.58 *μ*g/ml in the two cell types, respectively, while there was little inhibitory effect on A549, HT-29, and MCF-7 cells (Figure 1(a)). Hence, HepG2 cells were chosen for further experiments examining the anticancer effect of NnV. NnV inhibited the proliferation of HepG2 cells in a dose-dependent manner, while showing no toxicity in normal cells at the same concentrations (Figure 1(b) and Supplementary Figure  1, in Supplementary Material available online at https://doi.org/10.1155/2017/2752716). To confirm whether the antiproliferative effect of NnV is associated with apoptosis, cell cycle analysis using flow cytometry was carried out. The subG1 population (indicating induction of apoptosis) in HepG2 cells was increased after treatment with NnV in a dose-dependent manner from 3.05% to 69.44% (Figure 1(c)). In addition, NnV-treated HepG2 cells were observed to undergo nuclear fragmentation and cytoplasmic shrinkage via Hoechst staining (Figure 1(d)). These results suggest that NnV strongly inhibits the growth of HepG2 cells through induction of apoptotic cell death, but not that of normal cells.

### 3.2. NnV Regulates Caspase Activation and Pro/Antiapoptotic Protein Expression in HepG2 Cells

**Figure 2:**
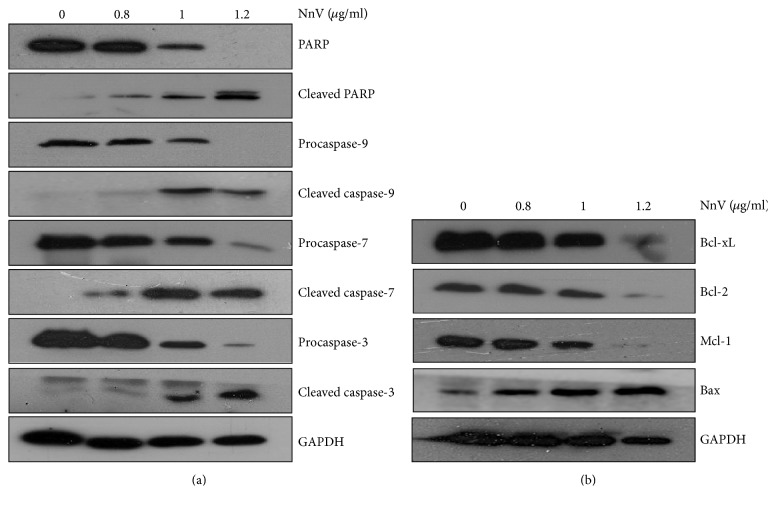
Effects of NnV on Bcl-2 family proteins and caspases in HepG2 cells. (a) HepG2 cells were treated with NnV (0, 0.8, 1, and 1.2 *μ*g/ml). The levels of apoptosis-related proteins were analyzed by western blotting using specific monoclonal antibodies. (b) The expressions of Bcl-2 family proteins were analyzed by western blotting using specific monoclonal antibodies. GAPDH was used as a loading control.

The apoptotic process is mainly executed by caspases. Caspase-9 is the essential initiator and caspase-3 and caspase-7 are the effectors required for mitochondrial apoptosis [28]. To explore the molecular mechanisms of action of NnV on apoptotic HepG2 cell death, we measured the expressions of key caspase proteins. As shown in Figure 2(a), the expressions of procaspase-3, procaspase-7, and procaspase-9 were dramatically decreased, while cleaved caspase-3, caspase-7, and caspase-9 were increased by NnV treatment in a dose-dependent manner. Furthermore, it is well known that cleavage of poly-ADP ribose polymerase (PARP) by activation of caspase-3 plays a critical role in apoptosis through blocking DNA repair [29]. As expected, the expression of full-length PARP was decreased and that of cleaved PARP was increased in NnV-treated HepG2 cells. The expression levels of Bcl-xL, Bcl-2, and Mcl-1 were also significantly reduced in NnV-treated HepG2 cells, whereas the level of Bax was considerably elevated (Figure 2(b)). These results indicate that NnV induces apoptotic cell death in HepG2 cells.

### 3.3. NnV Inhibits the PI3K/Akt/mTOR Signaling Pathway in HepG2 Cells

**Figure 3:**
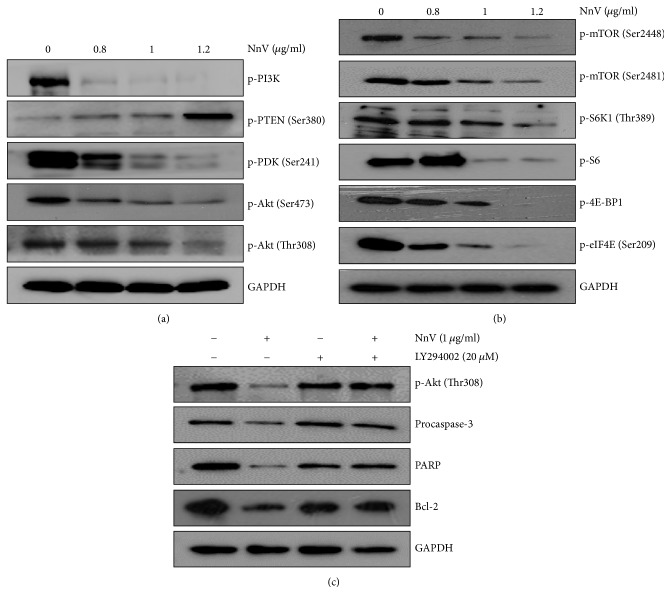
Effects of NnV on PI3K/Akt pathway in HepG2 cells. (a) The levels of p-PI3K, p-PTEN, p-PDK1, and p-Akt were analyzed by western blotting using specific monoclonal antibodies. GAPDH was used as a loading control. (b) The levels of p-mTOR, p-p70S6K, p-S6K, p-4E-BP1, and p-eIF4E were analyzed by western blotting using specific monoclonal antibodies. (c) HepG2 cells were pretreated with LY294002 (20 *μ*M) for 1 h and then treated with NnV.

The PI3K/Akt/mTOR signaling pathway is overexpressed or activated in various tumors and may inhibit apoptotic cell death and enhance survival and resistance to chemotherapy of cancer cells [30, 31]. To elucidate whether NnV-induced apoptosis is associated with the PI3K/Akt/mTOR signaling pathway, alterations in the expression of its signaling proteins were detected. In HepG2 cells, NnV treatment decreased the levels of p-PI3K, p-PDK1 (Ser241), and p-Akt (Thr308), whereas it increased the phosphorylation of phosphatase and tensin homolog (PTEN), a negative regulator of the PI3K/Akt pathway (Figure 3(a)). Additionally, NnV decreased the expression of p-mTOR (Ser2448), p-S6K1 (Thr389), and p-4E-BP1 (Thr37/46) (Figure 3(b)). NnV also suppressed p-S6 (downstream of S6K1) and p-eIF4E (a substrate of 4E-BP1) in a dose-dependent manner. PI3K/Akt-mediated mTOR1 inhibition promotes negative feedback loops, which lead to cancer cell proliferation, migration, and survival through activation of Akt at Ser473 [32, 33]. Interestingly, NnV inhibited phosphorylation of Akt at Ser473 and p-mTOR at Ser2481 (indicator of mTOR2 activation) (Figures 3(a) and 3(b)). To confirm whether PI3K/Akt/mTOR signaling is necessary for the induction of apoptosis by NnV, HepG2 cells were pretreated with LY294002 (an Akt inhibitor). Cotreatment with LY294002 and NnV significantly increased the expressions of procaspase-3, PARP, and Bcl-2, indicating that the anticancer effect of NnV is closely associated with inactivation of the PI3K/Akt/mTOR signaling pathway (Figure 3(c)).

### 3.4. NnV Inhibits Hepatic Tumor Growth In Vivo

**Figure 4:**
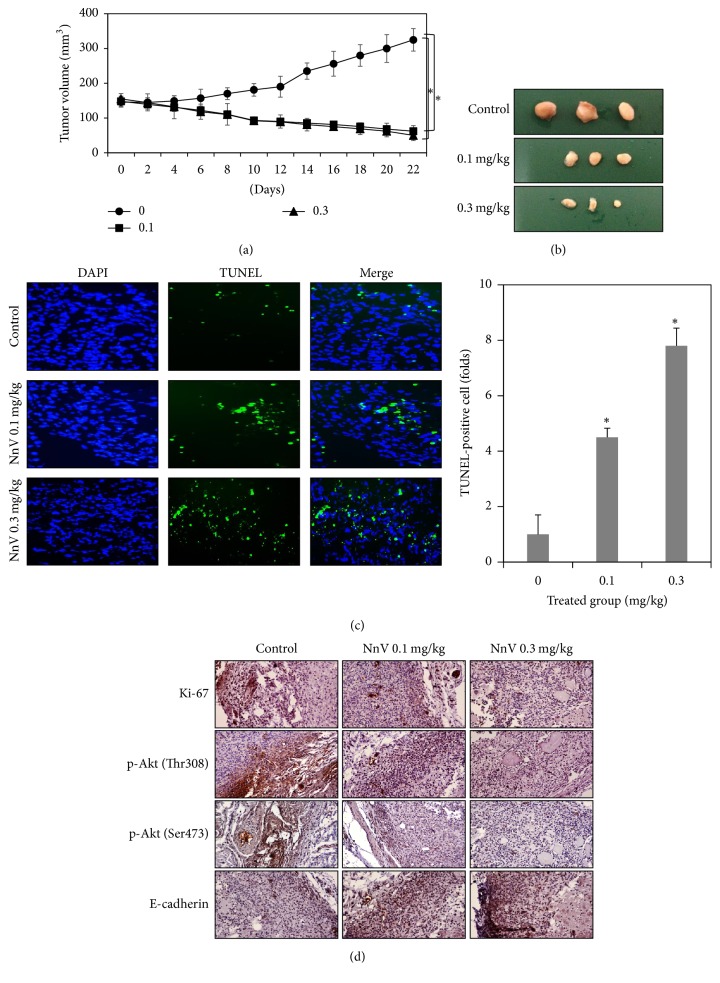
Effects of NnV on HepG2 tumor growth and induction of apoptosis. (a) Mice were orthotopically implanted with HepG2 cells and treated with NnV (0.1 and 0.3 mg/kg, I.P) for 20 day as described in Materials and Methods. Tumor sizes were measured by microcalipers every two days and average tumor volumes were representing the total tumor volumes per mouse. (b) On day 22, mice were sacrificed and the tumors were collected. Representative photographs of tumors are shown. (c) Induction of apoptosis was analyzed by TUNEL assay. As shown in the right panel, number of TUNEL-positive cells presented as folds compared with control group. (d) Tumor tissues were analyzed by immunohistochemistry to identify Ki-67 and p-Akt at Ser473 and Thr308 and E-cadherin expressions (×200 objective magnification). The results are expressed as the mean ± SD. ^*∗*^*p* < 0.01 was considered to indicate statistical significance compared with control group.

The in vitro study demonstrated that NnV has an anticancer effect on HepG2 cells through dual inhibition of Akt and mTOR. Therefore, the impact of NnV on hepatic tumor growth was assessed using a xenograft mouse model. After tumor volume increased to over 100 mm^3^, the tumor-bearing mice were treated with NnV (0.1 and 0.3 mg/kg) once every two days for 20 days. Tumor volume in the control group increased throughout the experimental period, whereas tumor volume in NnV-treated mice significantly decreased from the start of treatment until the end of the experiment (Figure 4(a)). On the 22nd day, the tumor volume reduction rates were 81.9% and 82.8% in the mice treated with NnV at doses of 0.1 and 0.3 mg/kg, respectively. Tumor growth in NnV-treated mice was also dramatically reduced (Figure 4(b)). During the experimental period, we observed no death or side effects in all experimental mice. Although the levels of AST, ALT, and CK were increased in NnV-treated groups, their level was normal. Furthermore, there were no significant changes in body weight and histological alteration in heart or liver tissue in NnV-treated mice, indicating that effective doses of NnV are relatively safe (Supplement Figure  2).

### 3.5. NnV Induces Apoptosis Partly through Akt and mTOR Signaling in the Xenograft Model

To confirm the mechanism of the anticancer effect exerted by NnV in xenograft tissues, we performed the TUNEL and IHC assays. TUNEL-positive cells were more prevalent in NnV-treated tumor tissue in a dose-dependent manner, and their numbers were significantly greater, by 4.5- and 7.8-fold, respectively, in the NnV 0.1 and 0.3 mg/kg groups (Figure 4(c)). The IHC assay demonstrated that the expression of Ki-67 was remarkably reduced in NnV-treated tumor tissue (Figure 4(b)). The expression of p-Akt at Thr308 and Ser473 was also diminished in NnV-treated tumor tissue. Interestingly, NnV enhanced E-cadherin expression, indicating that NnV may affect specialized cell-cell connections and cell-cell matrix interactions.

### 3.6. The Anticancer Effect of NnV Is Mainly Associated with Metalloprotease

**Figure 5:**
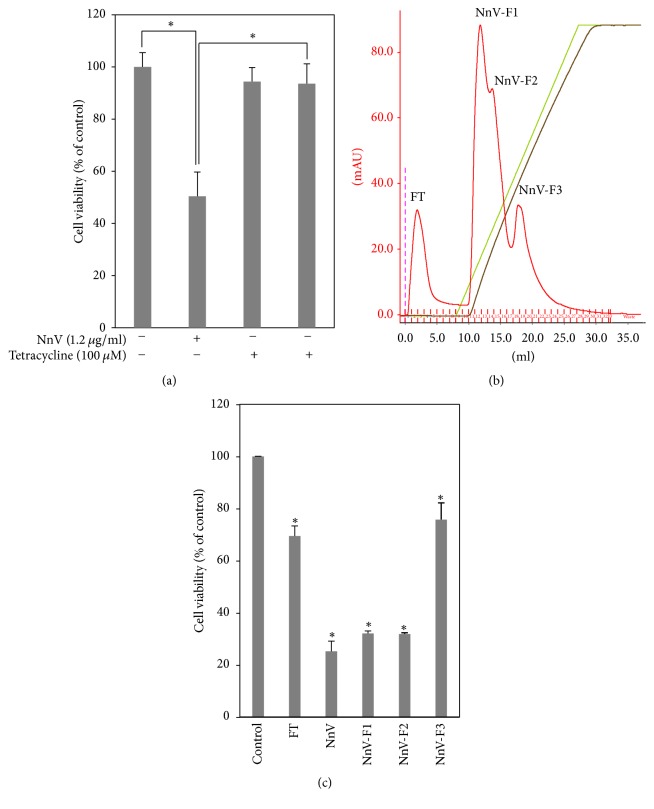
Isolation of anticancer component from NnV. (a) NnV preincubated with tetracycline (Tetra) 150 *μ*M for 3 h was treated in HepG2 cells for 24 h and then MTT assay was performed. (b) Crude venom was dissolved in 10 mM Tris-HCl (pH 7.8) buffer and centrifuged for 30 min at 13,000 ×g. The supernatant was introduced in a DEAE column and the proteins were eluted with a NaCl linear gradient of 0 to 80% at flow rate of 1 ml/min. (c) All fractions were evaluated for cytotoxicity in HepG2 cells.

To confirm the bioactive component of the anticancer effect of NnV, we performed a cytotoxicity test using tetracycline (a metalloprotease inhibitor, Tetra). NnV induced 50% cytotoxicity, whereas treatment with Tetra produced an almost full recovery of cell viability, indicating that the anticancer effect of NnV is closely associated with metalloprotease (Figure 5(a)). Hence, we tried to isolate the bioactive component using a DEAE ion exchange column. We observed three peaks, each of which was assessed by MTT assay (Figure 5(b)). That showed different results in that the viability of HepG2 cells was inhibited strongly by NnV-F1 and NnV-F2, but not NnV-F3 (Figure 5(c)). NnV-F1 and NnV-F2 induced cytotoxicity at levels comparable to that of crude venom (NnV), but F3 induced less cytotoxicity.

## 4. Discussion

There have been tremendous efforts by many investigators to discover novel components derived from the venom of various animals and to elucidate their modes of action, with the aim of developing therapeutic medicines, especially for the treatment of cancer. Jellyfish is a very interesting source owing to the high sensitivity and selectivity of its venom. As jellyfish blooms have increased in various parts of the world, there has been increased interest in the beneficial use of jellyfish venom, including its antiarthritic, antihypertensive, immunostimulatory, and insecticidal effects [21–23, 34, 35]. However, little is known about the beneficial effects of NnV. The present study investigated the anticancer therapeutic potential and mechanisms of action of NnV for the first time. Various animal venoms have anticancer effects as demonstrated by inhibition of cell proliferation, migration, invasion, apoptosis, and angiogenesis. According to reports on anticancer effect of bee venom, the IC_50_ values were 14.2, 6.3, and 6.1 *μ*g/ml in LNCaP, DU145, and PC-3 cells, respectively, after 72 h of treatment [36]. Another group reported that bee venom inhibited lung cancer cell growth at doses of 2.91 and 3.14 *μ*g/ml in A549 and NCI-H460 cells, respectively [37]. Furthermore, an effective dose of bufalin (derived from toad skin and parotid venom) in pancreatic cancer cells was 0.1 *μ*M (=3.865 *μ*g/ml), and ruviprase, a peptide purified from Russell's viper venom, induced apoptotic MCF-7 breast cancer cell death at 4 *μ*g/ml [38, 39]. The present findings showed that NnV selectively inhibits the proliferation of HepG2 cells at doses of 0.8–1.2 *μ*g/ml through induction of apoptotic cell death. Additionally, it is not toxic to normal hepatic epithelial cells, fibroblasts, and keratinocytes at the same concentrations. We did not observe any toxic or adverse effects in liver and heart tissues, such as changes in the level of function-related enzymes, findings on gross autopsy, or histopathologic changes in vivo. Therefore, the anticancer activity of NnV is as competitive as that of any other venoms.

To understand how NnV induces apoptosis, we investigated the PI3K/Akt/mTOR signaling pathway. The PI3K/Akt/mTOR signaling pathway is one of the major pathways that regulates cell proliferation, migration, angiogenesis, and survival [40]. This pathway is thought to be the most frequently aberrant pathway in numerous cancers, affecting some 30–50% of tumors, making it an attractive target for anticancer drugs [41]. Pathological activation of its signaling can occur in multiple ways, with the most common being point mutations in PI3K, loss of PTEN function, and hyperactivation of Akt. Akt is overexpressed in up to to 71.5% of HCC samples, leading to invasion, metastasis, and vascularization of HCC [40]. Aberrant mTOR signaling has been detected in up to 47.5% of HCC cases and results in poor prognosis [42]. Therefore, the PI3K/AKT/mTOR signaling pathway is a suitable therapeutic target in HCC. NnV dramatically decreased phosphorylation of PI3K, PDK1, and Akt at Thr308, whereas it significantly upregulated the expression of PTEN. Furthermore, NnV inhibited the phosphorylation of mTOR, which is downstream of Akt. NnV suppressed the phosphorylation of S6K1 and 4E-BP1, two well-characterized direct substrates of mTOR1, in a dose-dependent manner. Consistently, NnV suppressed phosphorylation of S6 and eIF4E. Despite the inactivation of PI3K-mediated p-Akt (Thr308), mTOR1 inhibition still promotes feedback activation of mTOR2-driven phosphorylation of Akt at Ser473 [32, 33]. Inhibition of PI3K/Akt-mediated mTOR signaling affects mTOR1, but not mTOR2. A compensatory feedback loop results in activation of mTOR2-driven Akt phosphorylated at Ser473. Akt is both an upstream activator of mTOR1 and a downstream effector of mTOR2 [32, 42]. Therefore, dual inhibition of PI3K/Akt and mTOR is an effective strategy for targeting cancer cells. We examined whether NnV inhibited mTOR2 signaling. NnV repressed p-Akt at Ser473 (an mTOR2 target site) as well as p-mTOR at Ser2481 (an indicator of mTOR2 activation) in HepG2 cells. Additionally, inactivation of Akt subsequently induces the mitochondrial apoptotic pathway through alterations in the ratio of Bax to Bcl-2 and activation of caspase-3. The caspase cascade is a vital hallmark of apoptosis which results in regulation of pro-/antiapoptotic proteins (Bax, Bcl-2, Bcl-xL, and Mcl-1). The Bax/Bcl-2 ratio is critical for the induction of apoptosis and determines whether or not cells will undergo apoptosis [43]. NnV treatment resulted in decreases in the levels of the antiapoptotic proteins Bcl-2, Bcl-xL, and Mcl-1 but led to an increase in the level of the proapoptotic protein Bax. The Bax/Bcl-2 ratio was dramatically increased from 1 to 134 by NnV treatment, leading to activation of caspase-3, caspase-7, and caspase-9 and inactivation of PARP. These results indicate that NnV promotes apoptosis through increasing the Bax/Bcl-2 ratio and subsequent activation of caspases.

We further conducted an in vivo study using xenograft animal models. As expected, NnV strongly suppressed tumor growth in HepG2-bearing mice through induction of apoptotic HepG2 cell death. Compared with the control group, tumor volume in the NnV-treated groups decreased by nearly 80%. The NnV-treated groups showed a remarkable reduction in the expression of Ki-67- (indicators of cellular proliferation) and an increase in the number of TUNEL-positive cells, indicating that NnV induces inhibition of cancer cell proliferation and apoptotic cell death. Based on our in vitro study, the effects of NnV on the Akt pathway were investigated in a HepG2 xenograft model. NnV exhibited dual inhibition of Akt at Thr 308 and Ser 473, which further suggests that NnV displays anticancer activity related to suppression of both Akt and mTOR. Interestingly, it was observed that NnV-treated tumor tissues are less diffused and more compact compared with tissues in the control group. To confirm whether NnV affects cell-cell density and cell-cell matrix interactions in tumor tissue, we assessed the expression of E-cadherin, which is an important protein involved in cell adhesion. The loss of its function promotes cellular motility and invasiveness by decreasing the strength of cellular adhesion, contributing to cancer cell progression and metastasis. NnV dose-dependently increased expression of E-cadherin in the HepG2 xenograft model, indicating that NnV may inhibit the migration and invasion of HepG2 cells by regulating E-cadherin. Further studies are necessary to demonstrate whether the novel anticancer effect of NnV is associated with regulation of the epithelial-to-mesenchymal transition (EMT).

Although this study demonstrates the anticancer effect of NnV, identifying the molecular mechanisms associated with this activity is difficult because full identification and characterization of individual components of venom have not been done. A previous study and unpublished proteomic data (being prepared for publication) demonstrated that NnV contains an abundant amount of metalloprotease-like enzymes [16]. According to proteomic profile, 150 toxin proteins were identified by 2-DE, including metalloprotease, serine protease inhibitor, phospholipase, and serine protease. Metalloproteases were major component in the* N. nomurai* venom with a proportion of 21% of the identified venom toxins. In other venoms (snakes and spiders), metalloprotease exerted inhibitory effects on cancer cell adhesion, proliferation, migration, invasion, and angiogenesis. Several studies reported that venom metalloprotease has potential anticancer effects [44]. The extracellular matrix (ECM) plays important roles in cell behavior and function, including growth, death, adhesion, and migration. These changes are mainly involved with interaction of integrin receptors, which can lead to apoptosis in cancer cells. Anoikis, a special type of apoptosis, is provoked by degradation of ECM components such as matrix metalloproteinases (MMP) [45]. Jararhagin, a purified snake venom metalloprotease from* Bothrops jararaca*, exhibited an inhibitory effect on cell adhesion and a cytotoxic effect on melanoma cells [46, 47]. TSV-DM, a metalloprotease from* Trimeresurus stejnegeri*, brought about inhibition of cell proliferation and induction of apoptotic cell death in ECV304 cells [48]. To investigate whether the anticancer effect of NnV is associated with metalloprotease, cell viability was assessed after preincubation with NnV and MMP inhibitors (tetracycline). NnV treatment alone caused 50% cytotoxicity in HepG2 cells, while cotreatment between NnV and Tetra had little effect on cell viability. Next, we tried to isolate the bioactive component that exerts the anticancer effect. We obtained three peaks, and each fraction was assessed for cytotoxicity in HepG2 cells. Compared with crude venom (NnV), F1 and F2 induced similar cytotoxicity, while NnV-F3 induced less cytotoxicity. To identify and characterize the active components that have an anticancer effect, sufficient amounts of NnV-F1 and NnV-F2 are necessary. However, NnV-F1 and NnV-F2 had very low protein concentrations, making it difficult to identify and characterize the active components. Other groups reported that identification of individual components of jellyfish venom is very difficult as a large amount of venom is necessary for isolation and characterization, and the venom has unstable properties and an insufficient amount of previously published data, unlike other types of venom (snake and scorpion) [17, 49]. Despite the potential of NnV, obtaining sufficient amounts of venom for further studies for identification and characterization remains a significant challenge.

In conclusion, although purification of anticancer component from NnV has not succeeded, we propose new insights into possibility of NnV in pharmacological intervention of cancer therapy. In the next study, we will establish a series of purification steps for the isolation of anticancer component and perform more detailed studies on the mechanisms of its action. By any measure, this is the first report to demonstrate that NnV has a selective anticancer effect, which acts partly through dual inhibition of PI3K/Akt and mTOR signaling in HepG2 cells and a xenograft model.

## Supplementary Material

Supplementary Figure 1: NnV induces cytotoxicities of several normal cell lines; WB-F344 (rat liver epithelial cell), HaCaT (human keratinocyte), HDF (Human dermal fibroblast) and H9C2 (rat cardiomyocyte). Each cells were treated with various concentrations of NnV for 24 h, and MTT assay was evaluated. The data shown are the mean ± SD of six independent experiments. ∗p<0.05 was considered to indicate statistical significance compared with non-treated controls.
Supplementary Figure 2: Toxicity evaluation of NnV in liver and heart tissues. To ensure the safety of NnV treatment, mice bearing HepG2 cells were sacrificed at the end of the experiment, and blood, liver and heart tissues were collected by routine procedure. (A) The parameters of liver and heart functions in control and NnV-treated mice were analyzed using Chemistry. (B) Body weights in the mice of control and NnV-treated group were checked during experiments. (C) Liver and heart tissues were evaluated by hematoxylin and eosin (H&E) staining (× 200 objective magnification). There are no histological change in NnV-treated groups.
