# Supplementary figures and images for: Anticancer Effect of Nemopilema nomurai Jellyfish Venom on HepG2 Cells and a Tumor Xenograft Animal Model
Source: Evid Based Complement Alternat Med. 2017 Jul 13;2017:2752716. doi: 10.1155/2017/2752716 (PMC5530421; doi:10.1155/2017/2752716)

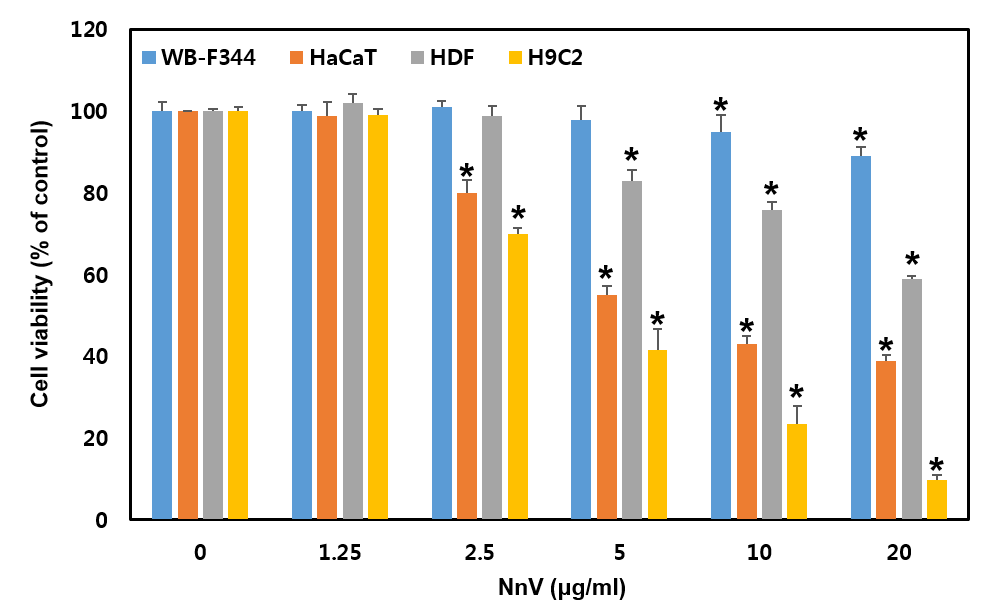

Supplement: Supplementary file 1 — Supplementary Figure 1: NnV induces cytotoxicities of several normal cell lines; WB-F344 (rat liver epithelial cell), HaCaT (human keratinocyte), HDF (Human dermal fibroblast) and H9C2 (rat cardiomyocyte). Each cells were treated with various concentrations of NnV for 24 h, and MTT assay was evaluated. The data shown are the mean ± SD of six independent experiments. ∗p<0.05 was considered to indicate statistical significance compared with non-treated controls. Supplementary Figure 2: Toxicity evaluation of NnV in liver and heart tissues. To ensure the safety of NnV treatment, mice bearing HepG2 cells were sacrificed at the end of the experiment, and blood, liver and heart tissues were collected by routine procedure. (A) The parameters of liver and heart functions in control and NnV-treated mice were analyzed using Chemistry. (B) Body weights in the mice of control and NnV-treated group were checked during experiments. (C) Liver and heart tissues were evaluated by hematoxylin and eosin (H&E) staining (× 200 objective magnification). There are no histological change in NnV-treated groups. [file 2752716.f1.zip › Supplementary Figure 1.tif]

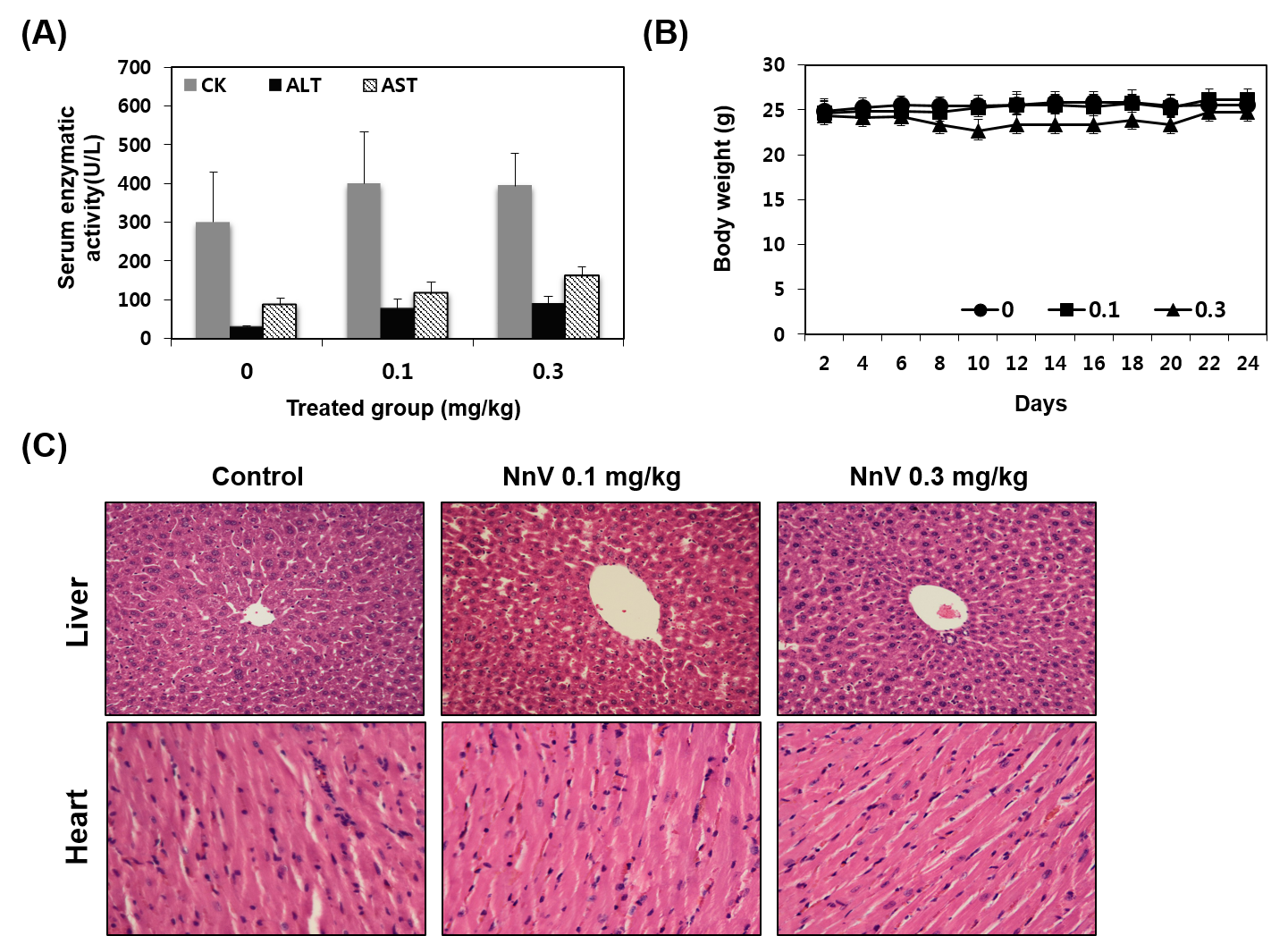

Supplement: Supplementary file 1 — Supplementary Figure 1: NnV induces cytotoxicities of several normal cell lines; WB-F344 (rat liver epithelial cell), HaCaT (human keratinocyte), HDF (Human dermal fibroblast) and H9C2 (rat cardiomyocyte). Each cells were treated with various concentrations of NnV for 24 h, and MTT assay was evaluated. The data shown are the mean ± SD of six independent experiments. ∗p<0.05 was considered to indicate statistical significance compared with non-treated controls. Supplementary Figure 2: Toxicity evaluation of NnV in liver and heart tissues. To ensure the safety of NnV treatment, mice bearing HepG2 cells were sacrificed at the end of the experiment, and blood, liver and heart tissues were collected by routine procedure. (A) The parameters of liver and heart functions in control and NnV-treated mice were analyzed using Chemistry. (B) Body weights in the mice of control and NnV-treated group were checked during experiments. (C) Liver and heart tissues were evaluated by hematoxylin and eosin (H&E) staining (× 200 objective magnification). There are no histological change in NnV-treated groups. [file 2752716.f1.zip › Supplementary Figure 2.tif]
